# Scoping review of community-wide screening to break the chain of TB transmission

**DOI:** 10.5588/ijtldopen.25.0807

**Published:** 2026-06-15

**Authors:** C.R Horsburgh, H.E. Jenkins, E.A. Kendall, H. Esmail, C.-Y. Chiang, L.F. White, G.B. Marks

**Affiliations:** 1 Departments of Global Health, Epidemiology and Medicine, Boston University Schools of Public Health and Medicine, Boston, MA, USA;; 2 Department of Biostatistics, Boston University Schools of Public Health, Boston, MA, USA;; 3 Division of Infectious Diseases, Department of Medicine, Johns Hopkins School of Medicine, Baltimore, MD, USA;; 4 MRC Clinical Trials Unit at University College London and WHO Collaborating Centre for TB Research and Innovation, UCL Centre for Global TB Research, Institute for Global Health, University College London, London, UK;; 5 Division of Pulmonary Medicine, Department of Internal Medicine, Wan Fang Hospital, Taipei Medical University, and Department of Internal Medicine, School of Medicine, College of Medicine, Taipei Medical University, Taipei, Taiwan;; 6 Burnet Institute, Melbourne, VIC, Australia.

**Keywords:** tuberculosis, TB prevalence, asymptomatic TB

## Abstract

**BACKGROUND:**

Active screening for TB may help address the global TB burden, but the optimal screening strategies are not defined. We have reviewed the effectiveness of community-wide active screening programmes to assess the characteristics associated with a reduction in TB.

**METHODS:**

We performed a scoping review of community-wide screening studies since 1945 and summarised those that either provided TB prevalence data at the start and end of screening or compared the screening programme to a control community where screening was not performed.

**RESULTS:**

Eight studies were identified in China, Czechoslovakia, Kenya, Malawi, South Africa, Uganda, Vietnam, Zambia, and Zimbabwe. Initial TB prevalence ranged from 84/100,000 to 1,014/100,000. Five studies demonstrated TB prevalence reductions from 44% to >80%, one showed evidence both for and against reduction, and two failed to demonstrate reduction. The successful studies were those that found, over the duration of the multi-year intervention, between 124% and 252% of the number of people with TB estimated to have been in the population at the beginning of the intervention, while unsuccessful studies found only 69%–83%.

**CONCLUSION:**

Reducing the force of infection can be accomplished by performing multiple cycles of community-wide screening to reach substantial numbers of community members with TB.

TB remains the single largest global infectious disease killer, and was responsible for 1.23 million deaths in 2024.^1^ The global prevalence of TB declined from 240 per 100,000 persons in 1990 to 183 per 100,000 persons in 2021, representing a 0.87% compound annual rate of decline.^2,3^ This slow rate of decline was more than offset by the increasing global population, so that the absolute number of people with TB increased from 12.7 to 14.5 million during this period. A serious coordinated global effort to control TB, the DOTS Strategy, was mounted by the WHO early in the 1990s, based on the care model of the International Union Against Tuberculosis and Lung Disease (The Union), developed by Karel Styblo and colleagues.^4^ This strategy has been updated and strengthened over the decades, but it continues to focus on persons with symptomatic TB seeking medical attention.

A major barrier to reducing the global TB burden is that many people with infectious TB do not have or do not recognise symptoms.^5^ Such ‘asymptomatic’ persons, whose TB is not diagnosed (or whose diagnosis is substantially delayed), continue to spread TB to others in their communities.^6–9^ In addition, even those who do recognise symptoms are frequently unsuccessful in obtaining medical care that results in treatment for TB due to inadequate primary care capacity and lack of availability of appropriate diagnostic tools. As a result, people with TB tend to remain undiagnosed and in the infectious pool for prolonged periods, resulting in ongoing transmission that sustains TB incidence and prevalence. Thus, it will be difficult to meaningfully interrupt the population-level cycle of TB transmission without finding and treating persons with TB outside of clinical diagnostic contexts.^10–15^ There is an ongoing debate on what population subgroups (e.g., household contacts of people with infectious TB, people with HIV or diabetes, people who are undernourished, smokers, or alcohol users) could be targeted for screening to identify people who generate transmission. However, the percentage of people with TB that have these conditions ranges from 3.5% to 20%, so even eliminating TB among one of these subgroups will leave a substantial proportion of TB undiagnosed and a substantial proportion of transmission unaddressed.^1,16^ Hence, community-wide approaches may be needed.

With the advent of effective chemotherapy, broad implementation of radiographic screening was used in many countries to find and treat people with TB. These programmes were associated with substantial reductions in the burden of TB, as outlined in the Supplementary Data. However, these programmes took place in communities that were quite different from those where TB now flourishes, making it difficult to translate this experience into guidance about when and how screening should be undertaken today.^17^ Therefore, in this review, we summarise community-wide TB screening programmes since 1960 that provide evidence about success or failure in reducing the burden of TB in order to describe the characteristics associated with reducing the force of TB infection in a community.

## METHODS

Using terms in English, we searched Medline, Embase and Community of Science (see Supplementary Data for details) for the years 1945–2024 following standard procedures for a scoping review.^18^ Titles that indicated that screening was for TB disease had abstracts independently reviewed by two authors (CRH and HEJ) to determine whether the effect of the intervention on the prevalence of TB was assessed. We also surveyed review articles on community-wide TB screening to identify any articles that our search might have missed. Those articles (and their Supplementary Data) were reviewed for inclusion. We also consulted our personal libraries for articles and historical summaries.

### Evidence of a reduction in the burden of TB

Articles that report the effectiveness of community-wide TB screening provide a variety of metrics, including the number of cases identified, effect on notifications, effect on incidence, effect on prevalence, effect on the Annual Rate of TB Infection (ARTI), or comparison with a control population. We only included studies that provided the TB prevalence before or during the intervention and either: 1) evaluated a change in prevalence over time; 2) evaluated prevalence after the intervention compared to a control community where the intervention was not performed; or 3) evaluated an effect on the ARTI over time; or 4) evaluated an effect on ARTI compared to a control community where the intervention was not performed.

### Characteristics of the studies and relationship to reduction in the TB burden

Tables 1 and 2 summarise the screening method, screening duration, total number of people screened, and total number diagnosed with TB, the total number of people diagnosed with TB during the intervention (those identified by the intervention itself plus those presenting to TB Programme outside of the intervention), and the ratio of this to the estimated number of people with TB in the population at the start of the intervention. We used the total number of people diagnosed with TB in order to capture the direct as well as the indirect effects of the intervention.

**Table 1.** Characteristics of community-wide TB screening studies and outcomes.

Country and dates	Study design	Duration	Screening strategy	Proportion of population screened and frequency	Outcome
Czechoslovakia, 1961–1964 (Kolin)	Prospective cohort with pre-post comparison	48 months	X-ray screening regardless of symptoms; sputum and/or laryngeal swabs for culture obtained from those with positive X-ray or symptoms	95% of population screened in two cycles	∼59% reduction in prevalence by 1965
Zimbabwe, 2006–2008 (DETECTB)	Prospective cohort comparing 2 screening strategies (mobile van versus door-to-door) with no control	34 months	Sputum obtained from persons with chronic cough >2 weeks followed by smear	77% of population screened overall in six cycles	∼41% reduction in prevalence compared to initial prevalence (both screening strategies combined)
Zambia and South Africa, 2006–2009 (ZAMSTAR)	Prospective cohort study comparing intervention to control communities	36 months	Sputum obtained regardless of symptoms, followed by smear	7.1% of population screened in five cycles	No detectable reduction in prevalence or ARTI compared to control community
Malawi, 2011–2018 (Blantyre)	Prospective cohort study with pre-post comparison	42 months	Sputum obtained from persons with chronic cough >2 weeks followed by smear	∼	∼80% reduction in prevalence by 2019
China, 2013–2015 (Dongchuan)	Retrospective cohort study compared to control communities	36 months	X-ray screening of persons with symptoms or in risk groups; sputum obtained from those with positive X-ray followed by smear	92% of population screened in three cycles	58% reduction in prevalence by 2017
Kenya and Uganda, 2013–2017 (SEARCH)	Prospective cohort study comparing intervention to control communities	36 months	Sputum obtained from persons with symptoms (or referred to TB clinic) followed by smear	90% of population screened in three cycles	21% reduction in TB incidence among HIV+ persons but no reduction in HIV– persons; 27% reduction in TB infection
Zambia and South Africa, 2013–2018 (PopART)	Prospective cohort study comparing intervention to control communities	48 months	Persons with symptoms of TB or history of contact with someone with TB either had smear or Xpert performed or were referred to the TB Programme for evaluation	60% of population screened in four cycles of annual screening	No detectable reduction in prevalence compared to control community
Vietnam, 2014–2017 (ACT3)	Prospective cohort study comparing intervention to control communities	36 months	Sputum obtained regardless of symptoms, followed by Xpert	80% screened each year in three cycles	44% reduction in prevalence compared to control community; some reduction in new infections (post-hoc)

ARTI = Annual Rate of TB Infection.

**Table 2.** Proportion of prevalent TB detected in community-wide TB screening studies. Details on derivation of parameters in Supplementary Data.

Country and study dates	Number of persons in intervention community	Initial TB prevalence per 100,000	Number of persons with TB in population at start of screening	Total number of persons with TB found during screening^A^	Duration of intervention (years)	Persons found as a proportion of number of initial persons with TB	Outcome
Czechoslovakia, 1961–1964 (Kolin)	100,418	233	234	590	4	252%	59% reduction in TB
Zimbabwe, 2006–2008 (DETECTB)	110,432 adults	650	718	1,517	2.8	211%	41% reduction in TB
Zambia and South Africa, 2006–2009 (ZAMSTAR)	704,957	1,000 (estimated)	7,050	5,864	3	83%	No detectable reduction in TB
Malawi, 2014–2018 (Blantyre)	130,173 adults	1,014	1,320	1,633	4	124%	∼80% reduction in TB
China, 2013–2015 (Dongchuan)	34,420	84	29	66	3	130%	58% reduction in TB
Kenya and Uganda, 2013–2017 (SEARCH)	79,817 adults	400	319	230	3	72%	No detectable reduction in TB but 27% reduction in infection
Zambia and South Africa, 2013–2018 (PopART)	400,000 adults	832	3,328	2,295	4	69%	No detectable reduction in TB
Vietnam, 2014–2017 (ACT3)	51,460 adults	389	200	407	3	204%	44% reduction in TB

A found through screening plus those with TB presenting to TB Programme outside of screening intervention.

## RESULTS

We identified 1,909 potential articles. Of these, we reviewed 59 abstracts and downloaded 56 articles. We identified eight examples of screening in nine countries, with varying strategies and success rates (Table 1). A PRISMA-ScR checklist and diagram are provided in the Supplementary Data. Descriptive summaries of these studies are below, in the order in which they were initiated. Table 2 and the Figure show the relationship between the study outcome and the number of people with TB identified and treated.

**Figure.**
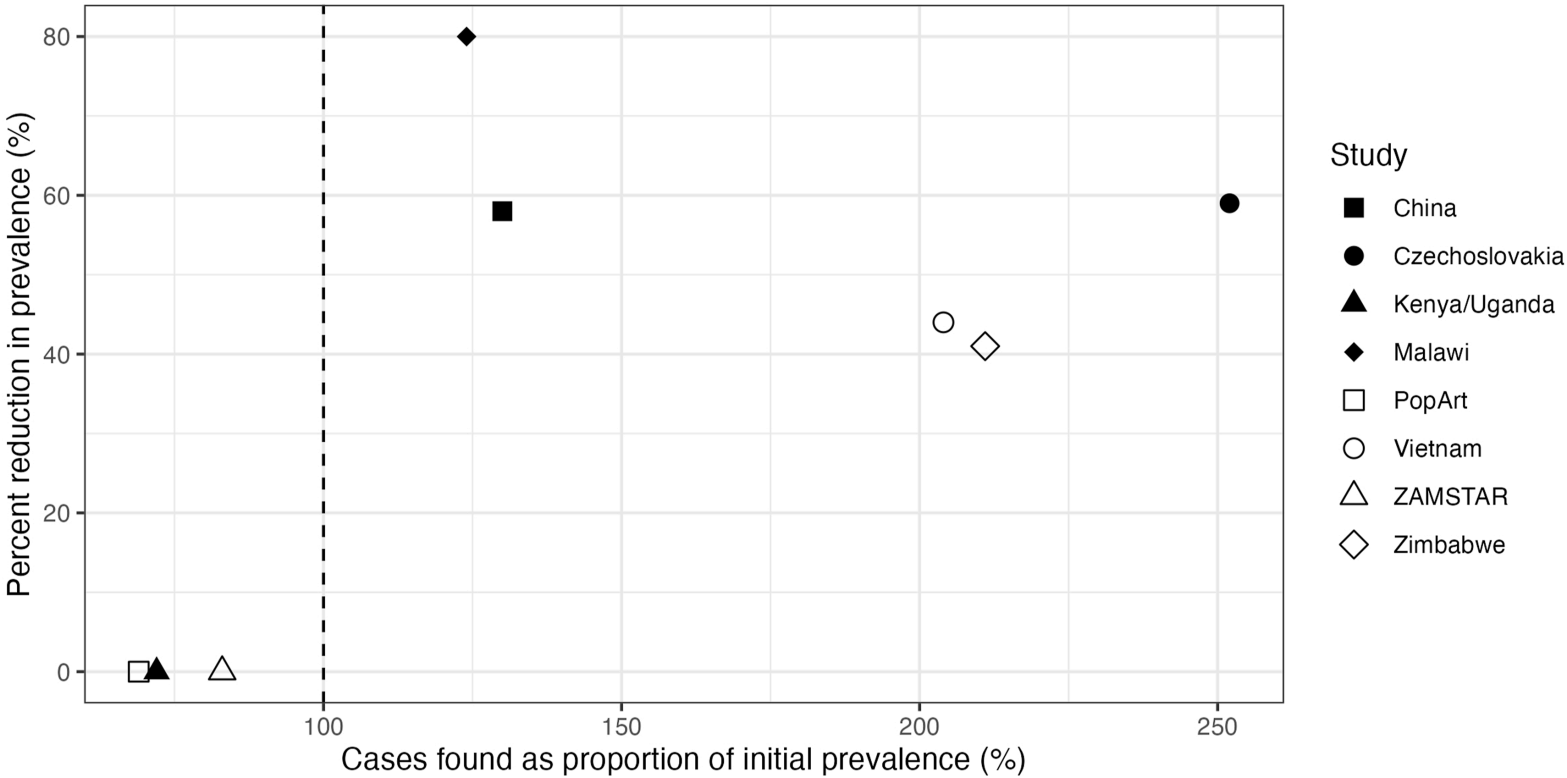
The relationship between the number of persons with TB diagnosed and treated and changes in TB prevalence.

*Czechoslovakia (Kolin), 1960–1964*.^19^ This project was undertaken in Kolin, a city of 100,498, to identify the best way to organise a TB control programme. Prior to initiation of the project, a preparation period of 18 months took place. The study used radiographic screening irrespective of symptoms to identify potentially infectious adults. Sputum was obtained from those with a radiograph suggestive of TB, and a diagnosis of TB was established using smear and culture of sputum and/or laryngeal swabs. The prevalence of smear- or culture-positive disease in 1961, at the start of the intervention, was thought to be 150/100,000, but when the first cycle of mass screening was performed in 1961 (with an estimated 95% participation), prevalence was found to be 233/100,000. The number of new cases of smear- or culture-positive TB, detected at mass photofluorographic screening, decreased from 97 in 1961 to 41 when the same population was rescreened in 1963 and half of those with TB had not felt sick enough to present for medical evaluation. After two cycles of screening that reached an estimated 95% of those over 14, a third city-wide radiographic screening revealed that prevalence in 1966 was 97/100,000, a 59% (48%–68%) decrease from 1961.^20^ The project continued through 1972, when prevalence was 56/100,000, demonstrating that the reduction continued for 8 years after the original intensive screening. New cases continued to occur, but these new cases largely resulted from reactivation of disease in persons previously diagnosed with inactive lesions.^21^ However, the effectiveness of isoniazid preventive therapy (IPT) in such persons was not demonstrated until 1976.^22^

*Zimbabwe (DETECTB), 2006–2008*.^23^ Adults with cough for ≥2 weeks were asked to provide sputum, and TB diagnoses were established by smear. The initial prevalence of culture-positive TB disease in the study area (residential suburbs of Harare) was 650/100,000. Screening for TB and HIV was performed at 6-month intervals for four cycles. In half of the randomly selected neighbourhoods, door-to-door visitation was used to find symptomatic persons, while in the other half a mobile van visited neighbourhoods and residents were screened at the van. The adult population eligible for screening comprised 110,432 adults and over the course of the study 10,177 adults (9.2%) reported cough for >2 weeks and were screened by obtaining sputum for smear microscopy. After five cycles of screening, the overall prevalence of culture-positive TB declined from 650/100,000 to 370/100,000; (adjusted risk ratio 0.59, 95% confidence interval [CI]: 0.40–0.89, *P* = 0.011). Mobile van screening was more effective than door-to-door visits (RR 1.48, 95% CI: 1.11–1.96, *P* = 00087).^24^

*Zambia and South Africa (ZAMSTAR), 2006–2009.*^25^ This study included community mobilisation with dramatisations, megaphone announcements, community meetings, leafleting with information about TB and the availability of the intervention, and, in half of the population, household contact screening for TB and HIV. The TB intervention consisted of providing increased access to sputum screening, but visitation to sputum collection centres was voluntary. A TB diagnosis was established using smear and culture. In a factorial design, neighbourhoods had either home visitation or community-level enhanced case finding (ECF) (community mobilisation with increased access to smear microscopy) or both, while a fourth set of communities only screened persons presenting to clinic with symptoms (control group). Screening was performed at 6-month intervals for six cycles with a prevalence survey performed in the final cycle. The adjusted prevalence ratio (PR) for the comparison of ECF versus non-ECF groups was 1.09 (95% CI: 0.86–1.40) and of household versus non-household intervention groups was 0.82 (0.64–1.04). The incidence of TB infection was measured in a cohort of 8,809 children, followed up for a median of 4 years; the adjusted rate ratio of incident TB infection for ECF versus non-ECF groups was 1.36 (0.59–3.14) and for household versus non-household groups was 0.45 (0.20–1.05). Thus, there was no evidence of a statistically significant reduction in TB.

*Malawi (Blantyre), 2011–2018*.^26^ A community-wide active case-finding programme for both TB and HIV was performed in high-density residential suburbs of Blantyre with an adult population of 130,173 between 2011 and 2018. The intervention included household visits, upgrading of clinic smear and culture capacity, and an enhanced surveillance system. Five cycles of screening were performed by visiting all households in the target neighbourhoods and inviting residents to provide sputum or visit a health care facility; any resident with a cough was asked to provide sputum for smear examination; TB was diagnosed by smear or, for participants seen at health centres, culture if smear was negative. After 2014, the household screening visits were discontinued, but the other parts of the intervention persisted. Other neighbourhoods in Blantyre served as control communities. Prevalence of TB in Blantyre City was not assessed prior to the initiation of the household screening visits, but was assessed in a national prevalence survey in 2013–14 as 1014/100,000, while in 2019–2020 a prevalence survey was performed in Blantyre that demonstrated TB prevalence of 150–189/100,000, a reduction of more than 80%.^27^

*China (Dongchuan), 2013–2015.*^28^ A retrospective cohort study reported the results of active case finding in 10 randomly selected communities in Dongchuan County, Yunnan Province, compared to 136 communities in the county that did not implement active case finding. 92% of households were visited yearly by trained community health workers. Residents with positive TB symptoms (cough or expectoration for over 2 weeks, or haemoptysis) or in a high-risk population (age > 65, diabetes, HIV/AIDS, close contact, and history of TB) underwent chest X-ray. Those with TB symptoms, or with abnormal lung shadows on X-ray if performed for other indications, were requested to submit three sputum samples for smear; diagnosis was by smear or radiography. Control communities had TB passively identified only. TB prevalence in the intervention communities went from 83.8/100,000 in 2012 to 35.4/100,000 in 2017, and in the control communities TB prevalence went from 78.8/100,000 in 2012 to 63.7/100,000 in 2017 (odds ratio 0.41, 95% CI: 0.28–0.61, *P* < 0.02).

*Kenya and Uganda (SEARCH), 2013–2017*.^29^ This intervention focused primarily on identification and treatment of HIV, but TB was included in the screening process. Sixteen matched pairs of communities with a population of ∼150,000 adults were randomised to baseline HIV testing and TB screening plus annual HIV testing and TB screening (intervention) or baseline HIV and TB screening only (control). Based on reported TB prevalence in these areas, the estimated prevalence in the study communities was 400/100,000.^30^ In both intervention and control communities, the programme was initiated with ‘mobile, 2-week, multi-disease health campaigns under large tents in all communities during weekdays, evenings, and weekends in collaboration with local health units’. Persons with HIV were offered ART and IPT; persons with TB symptoms (cough >2 weeks) either had sputum obtained for smear examination or were referred to the TB programme for further evaluation.^31^ After three cycles of annual HIV testing and screening for TB, the incidence rate of TB at 3 years among HIV-infected persons was 59% lower in the intervention group than in the control group (relative rate, 0.41; 95% CI: 0.19–0.86) but the incidence rate of TB among HIV-uninfected persons did not differ significantly between the two groups (relative rate, 1.03; 95% CI: 0.64–1.64). Thus, decreases in TB among HIV-infected persons could have been attributable to increased TB diagnosis and/or increase ART implementation (Isoniazid Preventive Therapy administration was limited). Nonetheless, in a follow-up study including HIV-infected and HIV-uninfected people, 1-year cumulative incidence of TB infection in the intervention communities was reduced by 27% (adjusted risk ratio 0.73 [0.57–0.92]).^32^

*Zambia and South Africa (PopART), 2013–2018*.^33,34^ The adult population in the intervention communities was 689,000. TB prevalence in these communities in 2011 was 832/100,000.^25^ A combined HIV and TB screening and treatment trial was undertaken between 2014 and 2017. Community sensitisation was conducted prior to the start of the trial, including distribution of information leaflets, door-to-door provision of information and involvement of a Community Advisory Board. The PopART intervention was delivered through a door-to-door approach, with the goal of reaching all selected households and all adult community members approximately annually. However, only 60% of eligible participants were reached. Screening for TB was based on TB symptoms (cough for ≥2 weeks, unintentional weight loss ≥1.5 kg in the last month, current night sweats), or that a member of the household was currently on TB treatment; sputum was collected for either GeneXpert or smear or the participant was referred to the TB Programme for evaluation. No evidence of a difference was seen comparing the two intervention arms with the control arm (adjusted PR 1.14, 95% CI: 0.67–1.95, *P* = 0.60) and TB prevalence for the intervention communities in 2017–2022 was 812/100,000, compared to the estimate of 832/100,000 in 2010.^35^ Moreover, a follow-up study of TB infection in adolescents and young adults revealed a slightly higher incidence rate ratio in the intervention communities, 1.45 (0.97–2.15).^36^

*Vietnam (ACT3), 2014–2018*.^37^ This study in Ca Mau Province was implemented after extensive consultation with stakeholders, including focus group discussions, meetings with community and political leaders, and interviews with local and national experts in health policy, TB control, and health promotion. The study used ability to produce sputum, regardless of symptoms or risk status, as its screening strategy to identify potentially infectious persons and a TB diagnosis was established with GeneXpert; positive Xpert results were confirmed with chest radiograph and culture. Intervention communities received three annual waves of house-to-house screening and control communities received no screening. The adult population screened in the intervention communities comprised 51,460 adults, and over the course of the study an average of 21,849 adults (42%) produced sputum each year. In the first year of the intervention, prevalence of disease was 389/100,000; in the second year it was 308/100,000 and in the third year it was 176/100,000. In the year following the intervention, prevalence of disease was 126/100,000 in the intervention communities and 226/100,000 in the control communities, prevalence ratio 0.56 (0.40–0.78; *P* < 0.001). A pre-planned ARTI study of children born in 2012 failed to show a significant decrease in the intervention communities compared to the control communities (PR 1.29, 0.70–2.36), but a subsequent ARTI study of randomly selected children born between 2004 and 2011 showed a significant ARTI decrease in the intervention communities (PR 0.50, 0.32–0.78). Subsequently, follow-up of persons screened in consecutive years also demonstrated a decrease in incident TB.^38^

## DISCUSSION

We identified eight screening studies that provided evidence about their effect on the force of infection. The most notable finding is that screening programmes that identified substantially more people with TB over the course of the intervention than were present at baseline (Kolin, DETECTB, Blantyre, Dongchuan and ACT3) had success, while the studies that found fewer (SEARCH, ZAMSTAR, PopART) showed either mixed results or failed to demonstrate reductions. The continued occurrence of new cases while screening is underway explains how more than 100% of initially prevalent people with TB can be identified and treated.

The screening strategies employed in the studies we examined were variable. Four studies screened with radiography; one screened all persons, while three limited radiography to those with symptoms. Four studies screened by obtaining sputum, followed by smear or Xpert; two screened all persons who could provide a specimen, while two others limited sputum collection to those with symptoms. In the studies from areas where TB prevalence was over 500/100,000, two successful studies (DETECTB and Blantyre) and one unsuccessful study (PopART) used symptomatic screening, while in the studies where prevalence was less than 500/100,000, the three successful studies (Kolin, Dongchuan, and ACT3) used symptom-agnostic screening. Thus, it may be that when TB prevalence is high, even limiting screening to those with symptoms can lead to significant disease reduction. In the two studies that followed up their populations after the initial phase of screening (Kolin and Blantyre), prevalence continued to decline for 8 and 6 years, respectively. This likely reflects the effects of both community sensitisation and upgrading of surveillance and diagnostic capacity.

It is also notable that all of the studies performed two or more cycles of screening. Repeated screening has the dual advantage of finding people with TB who were out of the area during the first screening and of identifying people who were incubating TB disease but had not clinically manifested it during the first cycle. Since it is well recognised that the 2 years following infection are the highest risk for disease, continued screening for at least 2 years would seem more likely to be able to interrupt the chain of transmission. The value of such rescreening has been demonstrated repeatedly.^39–41^ Additional characteristics that likely were important were engagement of the community, symptom-agnostic screening, and wide screening coverage; for example, in Kolin, only two cycles of screening had a substantial effect, as each cycle approached 95% coverage.

The WHO currently supports community-wide screening when the prevalence is above 500/100,000 based on cost-effectiveness considerations,^42^ but our results demonstrate that it can be effective even when prevalence is less than 100/100,000. As prevalence decreases, at some point TB disease screening will cease to be cost-effective, but more efficient screening tools may decrease this threshold.^43,44^ Several TB screening programmes in the Pacific Islands are already implementing combination disease and infection screening, although the success of such an approach remains to be demonstrated.^45,46^ Once a mass screening programme has led to substantial reductions in disease by identifying and curing persons actively spreading infection, a programme of preventive treatment of groups at increased risk of progression from infection to disease (such as those with untreated fibrotic lesions) could help to maintain the reduced force of infection.

Despite an association between the proportion of TB identified and reduction in the TB burden, we cannot conclude that this association can only be attributed to detection of TB by the intervention, as in two cases lesser declines in TB were also seen in the control populations (Dongchuan, ACT3). This suggests either that other factors were contributing to the observed decreases or that there was spillover from the intervention to the control communities, or both. It is important to note that five of the eight studies were performed in communities with a substantial HIV burden, and concurrent HIV screening and treatment could have provided additional impetus for reduction in the force of TB infection by decreasing susceptibility of the HIV-infected population.^29^ Moreover, the reports we reviewed did not provide data on cure/completion of TB treatment, without which no effect on TB burden would be expected. Nonetheless, most TB programmes achieve high rates of success in this metric.^1^

## CONCLUSION

In high-burden countries, a strategy of community-wide screening to identify and treat a substantial proportion of the population of persons with TB – and which is undertaken for several cycles – can lead to marked reductions in the TB burden. The first step in efforts to reduce TB needs to be engagement with the communities themselves to build momentum for a successful campaign. A second essential consideration is strengthening of the TB programme, since successful screening will generate many people who need treatment and this could overwhelm the capacity to do so. Once transmission has been substantially reduced, a programme of screening and treating TB infection should also be introduced.^47^ There are, of course, other important modalities for reducing transmission that need to be maximised, including better-tolerated regimens, effective infection control, and addressing comorbidities. However, these are unlikely, by themselves, to lead to the rapid reduction of prevalence that is needed. It will be challenging to eliminate TB as a global health threat, but we first need to ensure we are using the most effective strategies. New tools, such as better diagnostics and an effective vaccine will make the task easier, but there is no excuse for not making optimal use of our existing tools.

## Supplementary Material

## References

[1] World Health Organization. Global tuberculosis report 2025. Geneva: WHO, 2025.

[2] Murray CJL, Lopez AD. Global health statistics: a compendium of incidence, prevalence and mortality estimates for over 200 conditions. Cambridge, MA: Harvard University Press, 1996, Vol. 2.

[3] Yang H, Global, regional and national burden of tuberculosis and attributable risk factors for 204 countries and territories, 1990-2021: a systematic analysis for the Global Burden of Diseases 2021 study. BMC Public Health. 2024;24(1):11. 39529028 10.1186/s12889-024-20664-w PMC11552311

[4] Styblo K. Overview and epidemiological assessment of the current global tuberculosis situation: with an emphasis on tuberculosis control in developing countries. Bull Int Union Tuberc Lung Dis. 1988;63(2):39-44. 3224208

[5] Frascella B, Subclinical tuberculosis disease-A review and analysis of prevalence surveys to inform definitions, burden, associations, and screening methodology. Clin Infect Dis. 2021;73(3):e830-e841. 32936877 10.1093/cid/ciaa1402 PMC8326537

[6] Ryckman TS, Dowdy DW, Kendall EA. Infectious and clinical tuberculosis trajectories: Bayesian modeling with case finding implications. Proc Natl Acad Sci U S A. 2022;119(52):e2211045119. 36534797 10.1073/pnas.2211045119 PMC9907102

[7] Emery JC, Estimating the contribution of subclinical tuberculosis disease to transmission: an individual patient data analysis from prevalence surveys. Elife. 2023;12:e82469. 38109277 10.7554/eLife.82469 PMC10727500

[8] Stuck L, Prevalence of subclinical pulmonary tuberculosis in adults in community settings: an individual participant data meta-analysis. Lancet Infect Dis. 2024;24(7):726-736. 38490237 10.1016/S1473-3099(24)00011-2

[9] Horton KC, . Estimating the impact of tuberculosis pathways on transmission – what is the gap left by passive case finding? J Infect Dis. 2024;15(230):e1158-e1161. 10.1093/infdis/jiae390 PMC11566222 39106422

[10] Marks GB, . Epidemiological approach to ending tuberculosis in high-burden countries. Lancet. 2022;400(10365):1750-1752. 35934012 10.1016/S0140-6736(22)01433-7

[11] Wong EB. It is time to focus on asymptomatic tuberculosis. Clin Infect Dis. 2021;72(12):E1044-E1046. 33283223 10.1093/cid/ciaa1827 PMC8204778

[12] Ho J, Fox GJ, Marais BJ. Passive case finding for tuberculosis is not enough. Int J Mycobacteriol. 2016;5(4):374-378. 27931676 10.1016/j.ijmyco.2016.09.023

[13] Chaisson LH. Finding tuberculosis: more evidence for community-wide systematic screening. Clin Infect Dis. 2023;77(1):101-102. 37099289 10.1093/cid/ciad232

[14] Getahum H, Raviglione M. Active case-finding for TB in the community: time to act. Lancet. 2010;376:1205-1206. 20923714 10.1016/S0140-6736(10)61503-6

[15] Coleman M, Community-wide active case finding for tuberculosis: time to use the evidence we have. Trop Med Infect Dis. 2024;9:214. 39330903 10.3390/tropicalmed9090214 PMC11436250

[16] Kakaire R, Excess risk of tuberculosis infection among extra-household contacts of tuberculosis cases in an African city. Clin Infect Dis. 2021;73(9):e3438-e3445. 33064142 10.1093/cid/ciaa1556 PMC8563168

[17] Esmail H, Scaling-up symptom-agnostic, community-wide screening toward global tuberculosis elimination: opportunities, challenges, and lessons from history. Int J Infect Dis. 2025;155:107875. 40068708 10.1016/j.ijid.2025.107875

[18] Tricco AC, PRISMA extension for scoping reviews (PRISMA-ScR): checklist and explanation. Ann Intern Med. 2018;169(7):467-473. 30178033 10.7326/M18-0850

[19] Styblo K, Epidemiological and clinical study of tuberculosis in the district of Kolin, Czechoslovakia. Report for the first 4 years of the sttucy (1961-64). Bull WHO. 1967;37(6):819-874. 5301821 PMC2554234

[20] Krivinka R, Epidemiological and clinical study of tuberculosis in the district of Kolin, czechoslovakia. Second report (1965-1972). Bull WHO. 1974;51(1):59-60. 4549043 PMC2366257

[21] Steinbruck P, Tuberculosis risk in persons with ”fibrotic” x-ray lesions. Bull IUAT. 1972;47:135-059. 5077112

[22] Krebs A. The IUAT trial on isoniazid preventive treatment in persons with fibrotic lung lesions. Bull IUAT. 1976;51(1):193-201. 801115

[23] Corbett EL, Comparison of two active case-finding strategies for community-based diagnosis of symptomatic smear-positive tuberculosis and control of infectious tuberculosis in Harare, Zimbabwe (DETECTB): a cluster-randomised trial. Lancet. 2010;376(9748):1244-1253. 20923715 10.1016/S0140-6736(10)61425-0 PMC2956882

[24] Corbett EL, Prevalent infectious tuberculosis in Harare, Zimbabwe: burden, risk factors and implications for control. Int J Tuberc Lung Dis. 2009;13(10):1231-1237. 19793427 PMC3374846

[25] Ayles H, Effect of household and community interventions on the burden of tuberculosis in Southern Africa: the ZAMSTAR community-randomised trial. Lancet. 2013;382(9899):1183-1194. 23915882 10.1016/S0140-6736(13)61131-9

[26] Burke RM, Impact of community-wide tuberculosis active case finding and human immunodeficiency virus testing on tuberculosis trends in Malawi. Clin Infect Dis. 2023;77(1):94-100. 37099318 10.1093/cid/ciad238 PMC10320183

[27] Feasey HRA, Prevalence of bacteriologically-confirmed pulmonary tuberculosis in urban blsantyre, Malawi 2019-20: substantial decline compared to 2013-14 national survey. PLoS Glob Public Health. 2023;3:e0001911. 37862284 10.1371/journal.pgph.0001911 PMC10588852

[28] Chen J-O, Role of community-based active case finding in screening tuberculosis in Yunnan province of China. Infect Dis Poverty. 2019;8(1):92. 31661031 10.1186/s40249-019-0602-0 PMC6819334

[29] Havlir DV, HIV testing and treatment with the use of a community health approach in rural Africa. N Engl J Med. 2019;381(3):219-229. 31314966 10.1056/NEJMoa1809866 PMC6748325

[30] World Health Organization. National tuberculosis prevalence surveys 2007-2016. Geneva: WHO, 2021.

[31] Ssemundo E, Population-based active tuberculosis case finding during large-scale mobile HIV testing camparigns in rural Uganda. J Acquir Immune Defic Synd. 2016;73:e46-e50. 10.1097/QAI.0000000000001142 PMC6269148 27741032

[32] Marquez C, Community-wide universal HIV test and treat intervention reduces tuberculosis transdmission in rural Uganda: a cluster-randomized trial. Clin Infect Dis. 2024;78(6):1601-1607. 38226445 10.1093/cid/ciad776 PMC11175690

[33] Hayes R, HPTN 071 (PopART): rationale and design of a cluster-randomised trial of the population impact of an HIV combination prevention intervention including universal testing and treatment – a study protocol for a cluster randomised trial. Trials. 2014;15:57. 24524229 10.1186/1745-6215-15-57 PMC3929317

[34] Hayes RJ, Effect of universal testing and treatment on HIV incidence – HPTN 071 (PopART). N Engl J Med. 2019;381(3):207-218. 31314965 10.1056/NEJMoa1814556 PMC6587177

[35] Klinkenberg E, Tuberculosis prevalence after 4 years of population-wide systematic TB symptom screening and universal testing and treatment for HIV in the HPTN 071 (PopART) community-randomised trial in Zambia and South Africa: a cross-sectional survey (TREATS). PLoS Med. 2023;20(9):e1004278. 37682971 10.1371/journal.pmed.1004278 PMC10490889

[36] Shanaube K, The impact of a combined TB/HIV intervention on te incidence of TB infection among adolescents and young adults in th HPTN 017 (PopART) trrial communities in Zambia and Sounth Africa. PLoS Glob Public Health. 2023;3(7):e0001473. 37450474 10.1371/journal.pgph.0001473 PMC10348566

[37] Marks GB, Community-wide screening for tuberculosis in a high-prevalence setting. N Engl J Med. 2019;381(14):1347-1357. 31577876 10.1056/NEJMoa1902129

[38] Marks GB, A direct measure of tuberculosis incidence – effect of community screening. N Engl J Med. 2022;386(14):1380-1382. 35388676 10.1056/NEJMc2114176

[39] Okada K, Epidemiological impact of mass tuberculosis screening: a 2-year follow-up after a national prevalence survey. Int J Tuberc Lung Dis. 2012;16(12):1619-1624. 23131259 10.5588/ijtld.12.0201

[40] Dimairo M, The risk and timing of tuberculosis diagnosed in smear-negative TB suspects: a 12 month cohort study in Harare, Zimbabwe. PLoS One. 2010;5(7):e11849. 20676374 10.1371/journal.pone.0011849 PMC2911383

[41] Zhu P, Prognostic value of an abnormal chest X-ray result in predicting the development of tuberculosis. Nat Commun. 2025;16(1):9866. 41213917 10.1038/s41467-025-64834-9 PMC12603200

[42] World Health Organization. WHO consolidated guidelines on tuberculosis: module 2: screening: systematic screening for tuberculosis disease. Geneva: WHO, 2021. 33822560

[43] Veeken LD, Mind the clinic-community gap: re-evaluation of test performance and false positive results in community-wide tuberculosis screening. J Infect Dis. 2025;232(2):e242-e246. 40408778 10.1093/infdis/jiaf268 PMC12349932

[44] Andrews JR, Projecting the impact and costs of near point-of-care tuberculosis screening assays in community-based active case finding. Clin Infect Dis. 2025;17:ciaf395. 10.1093/cid/ciaf395 PMC12596382 40674508

[45] Brostrom RJ, TB-free ebeye: results from integrated TB and noncommunicable disease case finding in ebeye, Marshall Islands. J Clin Tuberc Other Mycobact Dis. 2024;35:100418. 38356926 10.1016/j.jctube.2024.100418 PMC10863304

[46] Coleman M, Population-wide active case finding and prevention for tuberculosis and leprosy elimination in Kiribati: the PEARL study protocol. BMJ Open. 2022;12(4):e055295. 10.1136/bmjopen-2021-055295 PMC9006843 35414551

[47] Migliori GB, The path to tuberculosis elimination: a renewed vision. Eur Respir J. 2023;61(6):2300499. 37080572 10.1183/13993003.00499-2023

