# Supplementary material for: Scoping review of community-wide screening to break the chain of TB transmission
Source: IJTLD Open. 2026 Jun 15;3(6):356–63. doi: 10.5588/ijtldopen.25.0807 (PMC13268051; doi:10.5588/ijtldopen.25.0807)
Supplement: Supplementary file 1 [file ijtldopen25-0807_supplementarydata1.pdf]

Supplement Contents:

1. Descriptions of early TB screening studies.

2. PRISMA-ScR Checklist and figure

3. Calculations of table parameters

1. Descriptions of early TB screening studies

Alaska 1947-60. At the end of World War Two the US government became concerned about the high rates of TB mortality in the Alaska Territory. A mass TB screening campaign was undertaken that reached 70% of the Alaskan population between 1946 and 1949. [1] An ambitious plan for construction of sanatoria was developed; however, the demonstration of effective chemotherapy in the early 1950s led instead to implementation of a broad chemotherapeutic program that was successful in reducing childhood TB infection to negligible levels over the following decade.[2] Although INH prophylaxis was studied in one district during this time, widespread prophylaxis was not initiated until later and is unlikely to have been a major factor in the reduction of TB in Alaska, which declined from and incidence of 500/100,000 in 1950 to 100/100,000 in 1960.[3]

South Africa 1948-94. Cape Town, South Africa instituted a Mass Miniature Radiography (MMR) screening campaign in 1948 that lasted until 1994. During this time screening was voluntary, but from 1952 to 1966 12-14% of the adult population was screened each year; this proportion decreased to 2% by the mid-1980s. It is notable that many of the cases were noted to be "asymptomatic and unsuspected". TB notifications decreased from 521/100,000 in 1952 to less than 30/100,000 in 1978.[4]

Denmark, 1950-52. In 1950, the Danish Tuberculosis Index was established as a co-operative undertaking of the National Health Service of Denmark and the WHO Tuberculosis Research Office. Under its auspices a mass radiography campaign was initiated, along with tuberculin testing and BCG vaccination, focusing on specific areas and age groups. Nearly 800,000 adults were examined, roughly 35% of the population. In the following four years the incidence of TB in adults dropped from roughly 75/100,000 to 25/100,000.[5]

The Netherlands, 1950-79. Community-wide radiographic screening for TB was conducted in the Netherlands, reaching 19% of the total population. The screening program was authorized under a "Population Screening Act for TB" in 1950 and was overseen by a government agency. Over time, participation and screening yields declined, while TB incidence fell from 159/100,000 in 1950 to 12.6/100,000in 1979.[6]

Japan 1951-98. In the 1950s Japan had a smear-positive TB rate of over 120 per 100,000. A mass miniature radiography program was implemented in 1951 and around 60% of the population over age 25 was screened each year. Of those detected on MMR, 35% were bacteriologically confirmed and referred for treatment. This program was associated with an annual decline of 11% per year and succeeded in reducing the TB rate to 35/100,000 by 1998.[7-9]

Australia 1952-80. Compulsory MMR of the entire adult population was implemented in Australia in 1952. During the first 20 years of the program, 1.5 million films were obtained. By 1976 the number of cases detected had decreased substantially so MMR screening was focused on men and smokers. Annual TB rates in Australia decreased from 54/100,000 in 1952 to 10.6/100,000 in 1980 and the MMR program was then continued only for selected subpopulations (smokers, migrants).[10,11]

Scotland, 1957. A one-time MMR screening program was undertaken in 1957 in Glasgow, a city of slightly over one million population with an estimated TB prevalence of greater than 400/100,000. In a 5-week period, 76% of the adult population received radiography and 2,565 people were diagnosed with TB requiring treatment. Over the ensuing 5-year period, the annual decline in TB notifications was 33% lower than in the pre-intervention interval, a statistically significant increase.[12]

References

1. Fortuine R. Must we all die? Alaska's enduring struggle with tuberculosis. University of Alaska Press, 2005, Anchorage, AK.
2. Kaplan GJ, Fraser RI, Comstock GW. Tuberculosis in Alaska, 1970. Am Rev Respir Dis 1972;105:920-926.
3. Chandler B. Alaska's ongoing journey with Tuberculosis. A brief history of tuberculosis in Alaska and considerations for future control. State of Alaska Epidemiology Bulletin 2017;19:1-8.
4. Hermans SM, Andrews JR, Bekker LG, Wood R. The mass miniature chest radiography programme in Cape Town, South Africa, 1948 - 1994: The impact of active tuberculosis case finding. S Afr Med J. 2016 Dec 1;106(12):1263-1269.
5. Groth-Petersen E, Knudsen J, Wilbek E. Epidemiological basis of tuberculosis eradication in an advanced country. Bull WHO 1959;21:5-49.
6. Esmail H, Miller C, Falzon D, et al. Scaling-up symptom-agnostic, community-wide screening toward global tuberculosis elimination: opportunities, challenges, and lessons learned from history. Int J Infect Dis 2025;155:107875.
7. Mori T. Recent Trends in Tuberculosis, Japan. Emerging infectious diseases. 2000; 6:566-8.
8. Ohmori M, Wada M, Uchimura K, Nishii K, Shirai Y, Aoki M. [Discussing the current situation of tuberculosis case-finding by mass miniature radiography in Japan]. Kekkaku. 2002; 77(4):329-39.
9. Ghotbi N, Nishimura S, Takatsuka N. Japan's national tuberculosis control strategies with economic considerations. Env Health Prev Med 2005;10:213-218.
10. Craven BR. Shoulders of Giants. Achievements in History's War on Tuberculosis. NSW, Australia: Blue Mountains Historical Society; 2010.
11. Pang SC. Chest radiography and tuberculosis case-finding in Western Australia. Resp Med 1998;92:198-202.
12. MacPherson P, Stagg HR, Schwalb A, et al. Impact of active case finding for tuberculosis with mass chest X-ray screening in Glasgow, Scotland, 1950-1963: an epidemiological analysis of historical data. PLoS Medicine 2024;21:e1004448.

2. PRISMA-ScR Checklist

Title:
Identifies Study as a Scoping Review - Yes

Abstract
Structured format - Yes

Introduction
Describes Rationale and specific Objectives - Yes

Methods
Protocol and registration: There is no specific protocol document for this review
Eligibility: Published studies, 1945-2024 in any language (only English language studies were identified)
Information sources: Medline, Embase and Community of Science. The rationale was that, as culture techniques for *M. tuberculosis* were not perfected until the late 1930s, there would not have been the possibility for studies to meet our criteria before the end of World War II.

Search: We ran the following searches: (("Community Health Services"[Mesh] OR "Community Health Services") AND ("Prevalence"[Mesh] OR Prevalence)) AND (("Tuberculosis, Pulmonary/epidemiology"[Mesh] OR "Tuberculosis, Pulmonary/prevention and control"[Mesh] OR "Mycobacterium tuberculosis"[Mesh] OR "Mycobacterium tuberculosis"))
and
(("Community Health Services"[Mesh] OR "Community Health Services" OR "Cross-Sectional Studies"[Mesh] OR "Cross-Sectional Studies") AND ("Prevalence"[Mesh] OR Prevalence)) AND (("Tuberculosis, Pulmonary/epidemiology"[Mesh] OR "Tuberculosis, Pulmonary/prevention and control"[Mesh] OR "Mycobacterium tuberculosis"[Mesh] OR "Mycobacterium tuberculosis"))

Titles that indicated that screening was for TB disease (not latent TB) had abstracts reviewed. Those articles (and supplementary materials) were reviewed for data abstraction. Articles were reviewed independently by two authors (CRH and HEJ).

Selection of sources of evidence: Articles that described an assessment of the effect of the screening intervention on the burden of TB were included.

Data charting process: The following variables were extracted from the articles themselves of from other published data about the community: country and date, screening strategy, proportion of population screened and frequency of screening, number of adults in the intervention community (and control community, where indicated), initial TB prevalence, number of people with TB identified each year and outcome of screening process on TB burden (success or failure).

Critical appraisal of sources of evidence: Not performed

Summary Measures: not calculated

Synthesis of results: Tables of extracted variables

Results

Selection of sources of evidence: Our search identified 1909 potential articles. Of these, 59 had abstracts reviewed and 37 articles were downloaded.

Characteristics of sources of evidence: See detailed descriptions in main article

Critical appraisal: See detailed descriptions in main article

Results: See Tables in article

Synthesis: Initial prevalence of TB ranged from 84/100,000 to 1014/100,000. All programs performed multiple rounds of screening. Four studies screened with radiography; one screened all persons, while 3 limited radiography to those with symptoms. Four studies screened by obtaining sputum, followed by smear or Xpert; two screened all persons who could provide a specimen, while two limited sputum collection to those with symptoms. Five studies demonstrated reductions in TB prevalence from 43% to 85%, one showed evidence both for and against reduction and two failed to demonstrate reduction. Successful screening was associated with annual identification of >30% of people with TB.

Discussion

Summary of evidence: Reduction of TB prevalence in high-burden communities can be accomplished by performing multiple rounds of community-wide screening that reach substantial numbers of community members with TB.

Limitations: Only two studies provided longer follow-up demonstrating that the reductions had been sustained.

Conclusions: Community-wide screening programs should engage the affected communities, strengthen TB treatment capacity and tailor their screening strategy to local conditions.

Funding

This work was supported by the US National Institutes of Health grants R35GM141821 and R01HL138728.

Supplemental Figure: PRISMA Flow Diagram

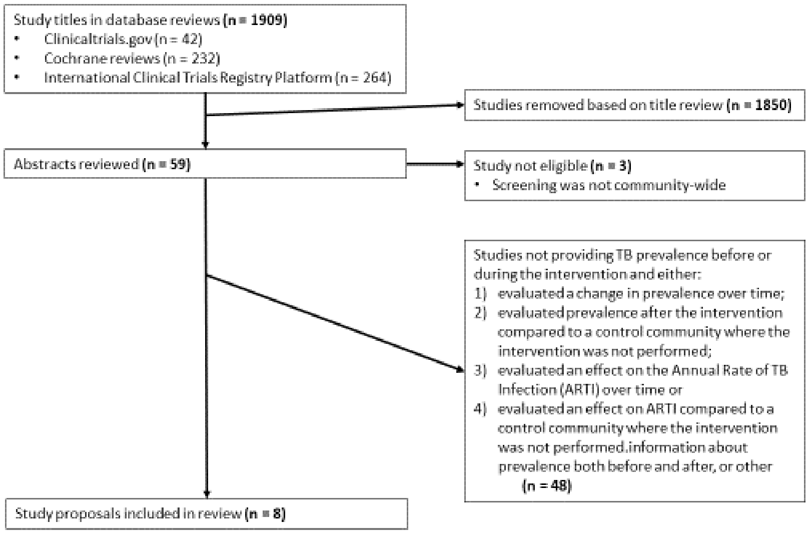

3. Calculations of table parameters

Czechoslovakia (Kolin). The intervention was implemented by the TB programme, so the number of cases identified in the article summarizes those found by screening and those presenting for clinical evaluation. During radiologic screening the authors estimate that 95% of the population was screened; thus, the prevalence estimate comes from a nearly complete population sample. Table 5 shows the number of TB cases identified in each of the four years, 1961-1964.[1]

Zimbabwe (DETECTB). The prevalence of culture-positive TB disease in the residential suburbs of Harare was 650/100,000, as determined by a 12% randomly selected sample of residents in the study area.[2] During the intervention period, 255 persons were found to be smear-positive by the mobile van teams, 137 were found by the house-to-house teams and 455 were subsequently confirmed to have TB (Table 2). Moreover, 670 additional residents were found to be smear-positive at clinics, a total of 1517 persons over a 34-month period.[2]

Zambia and South Africa (ZAMSTAR). This trial divided the study population into four parts, with one group receiving no intervention ("Clinic" only), one receiving "community-level enhanced case-finding (ECF; community mobilization and promotion of increased access to sputum examination using smear microscopy), one receiving Household Contact investigation at homes of persons with TB (HHC) and one receiving ECF and HHC. The TB prevalence at the outset was not known, but it was estimated by the authors to be ~1,000/100,000.[3] Because the results of TB screening are only presented for the ECF and ECF+HHC populations, these are the ones considered as the "Intervention group" for the purpose of our analysis. 1740 people with TB were identified by the ECF and ECF+HHC screening intervention and 4124 additional people with TB in the intervention communities were diagnosed in the clinics (p.1187).[4] This small number may be attributable to the voluntary referral process employed, in that persons identified as having evidence of TB at screening who were asymptomatic might well have not presented for formal evaluation and treatment.

Malawi (Blantyre). The intervention began in 2011 and the household visits continued until 2014, so the prevalence at initiation is not known. A prevalence survey was performed in Blantyre City in 2013-14 which revealed prevalence to be 1014/100,000.[5] Between 2014 and 2018, 1633 people were diagnosed with TB in the intervention communities by either smear, culture or Xpert (supplement, Table 2).[6] Thus, while we cannot determine the effect of the first three years of the intervention, we conclude that the other parts of the intervention (upgrading of clinic smear and culture capacity and an enhanced surveillance system) may have perpetuated any effects of the intervention.

China (Dongchuan). Prevalence of TB in the ten intervention communities in 2012 was reported to be 84/100,000.[7] The intervention was implemented by the TB programme, so the number of cases identified in the article summarizes those found by screening and those presenting for clinical evaluation. Sixty-six persons were diagnosed with active TB in the intervention communities (Figure 1).[7]

Kenya and Uganda (SEARCH). TB prevalence at the start of the study was estimated from a weighted average of rural prevalence in the Kenya 2015-16 and Uganda 2014-15 TB prevalence surveys.[8] The number of people with TB diagnosed through the intervention is not provided, but the total number of persons diagnosed with TB in the intervention communities during the study was estimated by multiplying the annual TB incidences provided in Figure 3 times the population in each year (provided in the supplement), stratified by HIV, yielding 230 people diagnosed with TB.[9]

Zambia and South Africa (PopART). Prevalence of TB in the study communities was assessed in a random sample prevalence survey in 2010, which found a TB prevalence of 832/100,000.[4] For the PopART intervention analysis presented here the two intervention arms (A and B) were combined. The initial population in arms A+B communities was ~689,000 overall and ~400,000 adults.[10] During the time of the intervention, each year a randomly selected group of adult residents was queried about whether they had been diagnosed with TB (and started of TB treatment) during the previous year. Over the 4 years of the intervention, 412 of 71,819 these adults in Groups A and B reported having had TB.[11] Applying this to the estimated adult population of adults in Groups A and B yields 2295 adults diagnosed with TB over the 4 year intervention. The final prevalence in Arm A communities was 910/100,000 and in Arm B communities was 700/100,000, giving a population weighted average of 813/100,000).[12]

Vietnam (ACT3). In the first year of the study a randomly selected prevalence survey was performed, demonstrating a TB prevalence in the intervention communities of 389/100,000.[13] Over the first 3 year intervention period (excluding the fourth year prevalence survey) 383 persons were diagnosed with TB, defined as "Xpert MTB positive" (Table S1) and an additional 24 people were diagnosed with TB outside of the survey.[13, 14]

References

1. Styblo K, Dankova D, Drapela J, et al. Epidemiological and clinical study of tuberculosis in the district of Kolin, Czechoslovakia. Report for the first 4 years of the sttucy (1961-64). Bull WHO 1967;37:819-74.
2. Corbett EL, Bandason T, Duong T, et al. Comparison of two active case-finding strategies for community-based diagnosis of symptomatic smear-positive tuberculosis and control of infectious tuberculosis in Harare, Zimbabwe (DETECTB): a cluster-randomised trial. Lancet 2010;376(9748):1244-53.
3. Ayles H, Schaap A, Nota A, et al. Prevalence of tuberculosis, HIV and respiratory symptoms in two Zambian communities: implications for tuberculosis control in the era of HIV. PLoS One 2009;4:e5602.
4. Ayles H, Muyoyeta M, Du Toit E, et al. Effect of household and community interventions on the burden of tuberculosis in southern Africa: the ZAMSTAR community-randomised trial. Lancet 2013;382(9899):1183-94.
5. Feasey HRA, Khundi M, Soko RN, et al. Prevalence of bacteriologically-confirmed pulmonary tuberculosis in urban Blantyre, Malawi 2019-20: substantial decline compared to 2013-14 national survey. PLoS Global Public Health 2023;3:e0001911.
6. Burke RM, Nliwasa M, Dodd PJ, et al. Impact of community-wide tuberculosis active case finding and human immunodeficiency virus testing on tuberculosis trends in Malawi. Clin Infect Dis 2023;77:94-100.
7. Chen J-O, Qiu Y-B, Rueda ZV, et al. Role of community-based active case finding in screening tuberculosis in Yunnan province of China. Infect Dis Poverty 2019;8:92.
8. WHO. National tuberculosis prevalence surveys 2007-2016. Geneva, Switzerland 2021.
9. Havlir DV, Balzer LB, Charlebois ED, et al. HIV testing and treatment with the use of a community health approach in rural Africa. N Engl J Med 2019;381:219-29.
10. Hayes R, Ayles H, Beyers N, et al. HPTN 071 (PopART): Rationale and design of a cluster-randomised trial of the population impact of an HIV combination prevention intervention including universal testing and treatment - a study protocol for a cluster randomised trial. Trials 2014;15:57.

11. Telisinghe L. Can universal testing and treatment for HIV and community-wide active case finding for tuberculosis control the African tuberculosis epidemic? Thesis submitted in accordance with the requirements for the degree of Doctor of Philosophy, University of London, 2024. Accessed at: https://researchonline.lshtm.ac.uk/id/eprint/4673624/
12. Klinkenberg E, Floyd S, Shanaube K, et al. Tuberculosis prevalence after 4 years of population-wide systematic TB symptom screening and universal testing and treatment for HIV in the HPTN 071 (PopART) community-randomised trial in Zambia and South Africa: A cross-sectional survey (TREATS). PLoS Med 2023;20(9):e1004278.
13. Marks GB, Nguyen NV, Nguyen PTB, et al. Community-wide Screening for Tuberculosis in a High-Prevalence Setting. N Engl J Med 2019;381(14):1347-1357.
14. Marks GB, Ho J, Nguyen PTB, et al. A direct measure of tuberculosis incidence – effect of community screening. N Engl J Med 2022;364:1380-82.
